# Electrostatic Screening, Acidic pH and Macromolecular Crowding Increase the Self-Assembly Efficiency of the Minute Virus of Mice Capsid In Vitro

**DOI:** 10.3390/v15051054

**Published:** 2023-04-25

**Authors:** Miguel Angel Fuertes, Diego López Mateos, Luis Valiente, Alicia Rodríguez Huete, Alejandro Valbuena, Mauricio G. Mateu

**Affiliations:** Centro de Biología Molecular “Severo Ochoa”, Universidad Autónoma de Madrid, Cantoblanco, 28049 Madrid, Spain

**Keywords:** virus, capsid, virus-like particle, nanocontainer, self-assembly, assembly efficiency, macromolecular crowding

## Abstract

The hollow protein capsids from a number of different viruses are being considered for multiple biomedical or nanotechnological applications. In order to improve the applied potential of a given viral capsid as a nanocarrier or nanocontainer, specific conditions must be found for achieving its faithful and efficient assembly in vitro. The small size, adequate physical properties and specialized biological functions of the capsids of parvoviruses such as the minute virus of mice (MVM) make them excellent choices as nanocarriers and nanocontainers. In this study we analyzed the effects of protein concentration, macromolecular crowding, temperature, pH, ionic strength, or a combination of some of those variables on the fidelity and efficiency of self-assembly of the MVM capsid in vitro. The results revealed that the in vitro reassembly of the MVM capsid is an efficient and faithful process. Under some conditions, up to ~40% of the starting virus capsids were reassembled in vitro as free, non aggregated, correctly assembled particles. These results open up the possibility of encapsidating different compounds in VP2-only capsids of MVM during its reassembly in vitro, and encourage the use of virus-like particles of MVM as nanocontainers.

## 1. Introduction

The protein capsids of many viruses are able to self-assemble from individual capsid building blocks (CBBs) under appropriate conditions. In vivo, some virus capsids are assembled without the participation of the viral nucleic acid, which is later encapsidated in an independent reaction; other virus capsids are co-assembled with the viral nucleic acid to directly yield virions (reviewed in [1,2,3]). However, it has long been known that, under certain conditions, even virus capsids that normally require the presence of the viral nucleic acid for assembly can be assembled in vitro in the absence of nucleic acid or any other biomacromolecule (e.g., the cowpea chlorotic mosaic virus (CCMV) capsid; [4,5,6]).

The finding of conditions for in vitro assembly of nucleic acid-free (empty) capsids from a number of different viruses has opened up the possibility of using them as nanocarriers or nanocontainers for a plethora of biomedical or nanotechnological applications. A general strategy involves the encapsidation of specific molecules or macromolecules during the in vitro assembly of either natural or engineered viral capsids (reviewed in [7,8,9]). The resulting virus-like particles (VLPs) containing appropriate cargos are being developed for specific chemotherapy by targeted drug delivery, contrast agents for biomedical imaging, nanobiosensors, nanocontainers for improved enzymatic reactions or the templated synthesis of inorganic nanoparticles, nanoscale materials, etc. (reviewed in [10,11,12]).

For papillomavirus capsids, conditions have been found for the very efficient (~60–100%) self-assembly of structurally correct capsids in vitro [13,14]. However, for many other virus capsids it was found that: (i) the conditions for in vitro assembly were different from those required for assembly inside a host cell (e.g., [15,16]); (ii) the assembly efficiency was low, if it was estimated at all; and (iii) aberrant and/or heterogeneous particles were frequently formed (e.g., [17,18,19]). For example, the capsid protein CA of the human immunodeficiency virus (HIV-1) is able to self-associate in vitro to form VLPs in the absence of any other macromolecule. However, to do so it requires either a very high, non-physiological salt concentration [15] that screens repulsive charges between capsid subunits [20,21], or the presence of a macromolecular crowding agent that increases the chemical activity of CA [22]. Moreover, CA tends to assemble into open tubes in vitro [15,20,22], although certain amino acid substitutions favor its assembly as truncated cone-shaped structures that resemble the authentic capsids formed in the maturing HIV-1 virion [23,24]. The identification of conditions for achieving the faithful and efficient assembly of specific viral capsids may lead to a deeper understanding of the foundations of self-assembly for virus capsids and non-viral protein complexes, and facilitate their production.

**Figure 1:**
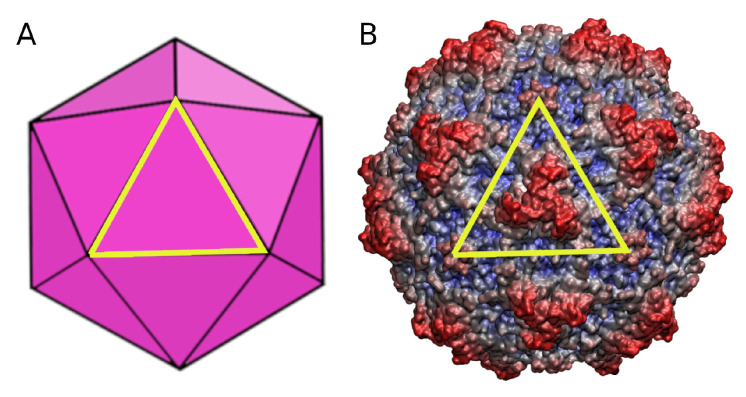
Scheme and structure of the MVM capsid. (**A**) Scheme of the icosahedral MVM capsid. (**B**) Surface representation of the atomic structure of the MVM capsid. The surface is color coded from blue to red as a function of the particle radius. In (**A**,**B**), the contour of a VP2 trimer (capsid building block) is indicated by a yellow triangle.

The capsids of parvoviruses, including the minute virus of mice (MVM) [25,26] may be useful for multiple biomedical or nanotechnological applications [27,28,29,30,31,32,33,34,35]. The icosahedral T = 1 MVM capsid (Figure 1) is one of the smallest and structurally simplest viral capsids known. It is only 25 nm in diameter and is made from a minimum number (60) of structurally equivalent protein subunits [36].

The authentic MVM capsid is made from two variants (VP2 and VP1) of the same capsid protein, which have an identical sequence and fold but differ in that VP1 has a longer N-terminal extension. VP2 alone is required for self-assembly, as the MVM capsid can be assembled from 60 identical VP2 subunits, both in transfected cells [37] and in the absence of any other macromolecule in vitro [38]. The MVM capsid is extremely robust and withstands high temperature, some harsh chemical treatments, and comparatively high mechanical forces [37,38]. In addition, MVM can be internalized in different cell types and contains signals that modulate its intracellular trafficking. During infection by MVM, pores in its capsid allow both the internalization and externalization of the single-stranded DNA genome and of peptide signals (reviewed in [39,40]).

In this study we have analyzed the effects of several different conditions on the fidelity and efficiency of self-assembly of the VP2-only MVM capsid in vitro. The results revealed that the in vitro reassembly of the MVM capsid is a remarkably faithful process, irrespective of the conditions used, and that its efficiency can be increased under certain conditions of pH, ionic strength, and/or macromolecular crowding.

## 2. Materials and Methods

### 2.1. Expression and Purification of MVM VLPs

MVM capsids made of 60 VP2 capsid protein subunits (termed here VLPs) were expressed, assembled in insect cells, and purified as described previously [37,38]. In essence, the procedure involved the following steps. A recombinant bacmid containing the VP2 protein gene (BM-VP2) was used to transfect H-5 cells. The progeny recombinant baculoviruses thus obtained were used to infect fresh H-5 cells. VP2 was expressed in infected cells from the recombinant baculovirus genome and formed VLPs inside the transfected cells. VLPs were purified by a process that involved two steps: (i) ultracentrifugation through a 20% sucrose cushion in TE buffer (50 mM Tris-HCl, 0.5 mM EDTA, 0.2% Triton X-100, pH = 8.0) at 16,000 rpm in an SW40 rotor (Beckman, Pasadena, CA, USA) for 21 h at 10 °C; and (ii) ultracentrifugation though a 10–40% sucrose gradient in phosphate-buffered saline (PBS) buffer (PBS; 8.1 mM Na_2_HPO_4_, 1.5 mM KH_2_PO_4_, 137 mM NaCl, 2.7 mM KCl, pH 7.6) at 30,000 rpm in an SW40 rotor for 5.5 h at 10 °C. Purified VLP suspensions were extensively dialyzed against PBS, concentrated by ultrafiltration using an Amicon-Ultra4 device of 100 kDa cutoff (Millipore, Burlington, MA, USA), and stored at 4 °C. VLP purity, integrity and monodispersity were analyzed by polyacrylamide gel electrophoresis and transmission electron microscopy (TEM). Because of the very large amount of VP2 and VLPs produced in insect cells, the two-step ultracentrifugation approach yielded highly pure preparations. MVM capsid protein concentration was determined by UV spectrophotometry.

### 2.2. Transmission Electron Microscopy (TEM)

Samples were deposited on ionized Formvar/carbon-coated copper grids (Electron Microscopy Sciences, Hatfield, PA, USA), incubated for 3 min and washed 3 times for 30 s with distilled water. Negative staining was carried out using incubation for 45 s in a 2% (weight/volume) uranyl acetate (Electron Microscopy Sciences) solution. The samples were then dried and visualized in a JEM-1010 electron microscope (JEOL) at magnifications ranging from 25,000× to 120,000×. Images were taken with a TemCam-F416 camera (TVIPS). Samples from each experiment were processed strictly in parallel, to allow a direct comparison of the data obtained.

### 2.3. VLP Disassembly and Reassembly

Purified native VLPs obtained from cells as described above were disassembled into free capsid building blocks. Purified VLP samples at a total protein concentration of 0.5–0.6 mg/mL were incubated at 25 °C in PBS pH = 7.6 in the presence of 3.5 M guanidinium chloride (GdmHCl) with agitation (350 rpm) in a Thermomixer Compact (Eppendorf, Hamburg, Germany) for a specified amount of time (between 45 min and 2.5 h, depending on the experiment) until VLP disassembly was nearly complete, as determined by TEM.

VLP reassembly from capsid building blocks obtained by the disassembly of purified VLPs as described above was triggered by fast dialysis of 150 µL samples for 12–25 min (unless otherwise noted) in Slide-A-Lyzer cassettes of 0.5 mL capacity and 10000 MWCO (Thermo Scientific, Waltham, MA, USA). Residual GdmHCl concentration after dialysis was estimated using a spectrophotometry-based approach [41]. Different capsid protein concentrations (from 0.02 to 0.65 mg/mL) and reassembly conditions, including pH, temperature, ionic strength, and the presence of a macromolecular crowding agent (Ficoll-70) were tested as described in Results. Buffers in which VLP reassembly was analyzed included PBS pH 6.0, 6.6, 7.2, 7.6 or 8.0, and PBS pH = 7.6 or 6.6, containing increasing NaCl concentrations (0.15, 0.5, 1.0 or 2.0 M) The actual pH of the reaction mixture was checked after completion of the assay. To analyze the effect of molecular crowding, Ficoll-70 (GE HealthCare, Little Chalfont, UK) was used. In every experiment, small aliquots were taken from samples incubated under different conditions at specified times, and the reassembly process was followed and quantitated by TEM.

### 2.4. VLP Reassembly Quantification and Reassembly Efficiency Calculation

The relative number of complete VLPs as a function of experimental conditions and time was estimated by counting the particles present in several (typically between 4 and 10) EM fields of a defined size (1.23 × 1.23 μm), and averaging the results. Particle counting was usually carried out using a home-made macro developed in Fiji [42]. In order to automate the analysis, a macro was written that uses the Template Matching plug-in [43], which allowed a quick and accurate counting of particles under the supervision of the user.

The reassembly efficiency was calculated as follows:

(1) $$R_{eff}\left(\%\right)=100\cdot \frac{N_{r}\left(D_{d}D_{r}\right)-\left(N_{d}D_{d}\right)}{N_{b}}$$

where *N_b_*, *N_d_* and *N_r_* are the average number of complete VLPs counted, respectively, before disassembly was triggered by adding GdmHCl (*N_b_*), just after the disassembly reaction was ended (*N_d_*, typically very close to 0) and after the reassembly reaction was ended (*N_r_*). *D_d_* is the dilution factor due to the addition of a volume of concentrated GdmHCl to trigger VLP disassembly. *D_r_* is the dilution factor due to the addition of a volume of a particular solution to determine VLP reassembly under certain specified conditions.

### 2.5. VLP Kinetic Stability Assay

The chemical stability of the MVM VLPs under different pH and ionic strength conditions in different buffers (150 mM Tris-HCl, 650 mM NaCl at pH = 7.6 or 6.8, or PBS at pH = 6.2 or 5.5) was tested by the addition of a GdmHCl solution to a final concentration of 3.75 M and incubation of the sample at 25 °C. Small aliquots were taken at different times and the percent remaining intact VLPs under each condition was estimated by TEM, as described above.

### 2.6. Molecular Graphics and Electrostatic Calculations

Models of MVM CBBs were built using the structure and symmetry matrices corresponding to the PDB entry 1Z14 [44]. Atomic charges were assigned as a function of the pH, and the potential under different conditions was calculated using the non-linear equation of Poisson–Boltzman [45] using the APBS tool [46]; the PQR file for the calculation was generated with the PDB2PQR tool [47]. Then, the VP2 trimer molecular surface was calculated, and the value of the potential was projected using a molecular visualization program, Chimera 1.11.2 version [48].

## 3. Results

### 3.1. Disassembly and Reassembly of MVM VLPs

Unless indicated otherwise, in this study the reassembly efficiency of MVM VP2-only capsids (VLPs) was defined in terms of the final yield of free VLPs assembled from capsid building blocks that had been obtained by disassembly of native VLPs. This is a demanding definition: even if 100% of the possible VLPs were correctly reassembled, the reassembly efficiency thus defined would be lower if some of them dissociated or became aggregated before the product was analyzed.

To determine the reassembly efficiency under each tested condition we used as a starting point a previously described experimental setup ([38]; see Methods). In our previous study, described in [38], purified, monodisperse VLPs of MVM at a chosen concentration were subjected to disassembly using controlled incubation at 25 °C in PBS pH = 7.6 containing 3.5 M GdmHCl. The reaction was left to proceed until no or very few complete or incomplete VLPs remained, as determined by transmission electron microscopy (TEM) and atomic force microscopy (AFM) imaging. In the same previous study [38], analysis using size exclusion chromatography revealed a shift from a single peak with the molecular weight corresponding to the intact VLP before disassembly to a single peak with the molecular weight corresponding to a VP2 trimer (195 kDa) after the disassembly reaction was terminated. In addition, height measurements using AFM confirmed that, after disassembly, only a few VLP fragments larger than trimers were present. Thus, the results of the previous study [38] indicated that, under the conditions tested, the VLPs were almost completely dissociated into VP2 trimers, the stable building blocks that form the MVM capsid [49,50]. Exactly the same VLP disassembly procedure described in [38] was used in the present study to determine the reassembly efficiency under different conditions. TEM imaging before (Figure 1A) and after disassembly (Figure 1B) confirmed that, as expected from our previous study using the same conditions, most of the original VLPs had been disassembled into small, unresolved subunits, and only very few larger capsid fragments remained.

**Figure 2:**
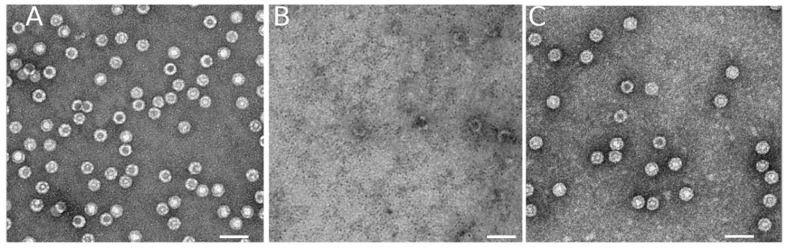
Disassembly and reassembly of MVM VLPs. Representative TEM images taken at the same magnification and total capsid protein concentration during a representative disassembly and reassembly experiment. (**A**) Purified VLPs prior to disassembly. (**B**) Viral capsid protein after the VLPs in the original sample were disassembled. (**C**) VLPs reassembled at 25 °C in PBS pH = 7.6. The scale bar corresponds to 50 nm.

To start the reassembly reaction, most of the GdmHCl was rapidly removed by dialysis under the conditions chosen for VLP reassembly (Figure 2C). Dialysis for 10–15 min reduced the GdmHCl concentration to a low value that did not impair reassembly. The reassembly efficiency under each set of conditions tested was calculated by counting complete VLPs in TEM images, as described in Materials and Methods. The number of free VLPs obtained after reassembly (Figure 2C) was divided by the number of original VLPs that were present before the disassembly reaction was started (Figure 2A). Before counting, the samples were diluted enough to avoid incorrect estimation of reassembly efficiencies due to grid saturation (Supplementary Figure S1). Incomplete or aberrant particles or reassembled VLP that formed aggregates, if present, were not considered in estimating the reassembly efficiency. To obtain statistically significant values, many EM fields similar to those in Figure 2 were counted in that way, and the results were averaged.

### 3.2. Effect of Viral Capsid Protein Concentration on VLP Reassembly Efficiency

**Figure 3:**
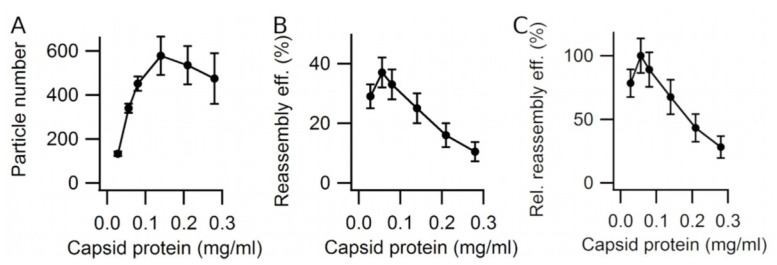
VLP reassembly efficiency as a function of protein concentration. Reassembly of VLPs was performed at 25 °C in PBS pH = 7.6, using different protein concentrations. Efficiency values were obtained as indicated in Methods by averaging the results of counting many EM grid fields. Vertical bars represent standard deviations. *t_f_* = 140 min. (**A**) Total number of reassembled VLPs. (**B**) Absolute reassembly efficiency. (**C**) Reassembly efficiency relative to the highest efficiency achieved (at 0.056 mg/mL).

The total number of free reassembled VLPs and the reassembly efficiency were estimated first as a function of capsid protein (VP2) concentration at 25 °C in PBS pH = 7.6 (Figure 3). Concentrations well above the critical concentration for assembly (<0.002 mg/mL; [38]) were used. The total number of free reassembled VLPs increased with the protein concentration until a plateau was reached at a relatively high protein concentration value (~0.15 mg/mL) (Figure 3A). In contrast, the reassembly efficiency increased at comparatively low protein concentrations only (up to ~0.06 mg/mL), but decreased linearly with protein concentrations from ~0.06 to ~0.3 mg/mL (Figure 3B,C). The maximum reassembly efficiency obtained under these conditions was 37 ± 5% (Figure 3B).

**Figure 4:**
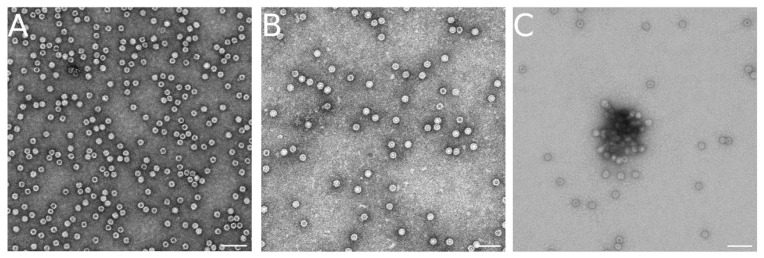
VLP aggregates formed during the reassembly reaction. Representative TEM images showing free recombinant VLPs purified from cells and kept at room temperature (**A**), free VLPs after in vitro disassembly and reassembly for 20 min (**B**), and a mixture of free and aggregated VLPs after disassembly, reassembly, and further incubation for a total of 3.3 h (**C**). Scale bars correspond to 100 nm.

The main reason behind this decrease in reassembly efficiency (defined as the ratio between the number of free VLPs obtained and the number of original VLPs) at the higher protein concentrations was traced to the formation of VLP aggregates (Figure 4). These aggregates became more abundant as the protein concentration was increased, as well as after longer reassembly times. TEM imaging of these aggregates indicated that they were formed by reassembled VLPs of the right size (25 nm diameter) and shape (Figure 4C). VLPs in those aggregates could not be counted, and were not considered when the reassembly efficiency was estimated. The results indicated that a maximum number of VLPs could be obtained at 0.15 mg/mL, at the cost of a large amount of capsid protein ending up in VLP aggregates. However, if one wished to maximize the number of free VLPs for a given amount of viral protein, one could choose a protein concentration of 0.06 mg/mL, at the cost of having to escalate the process in terms of reaction volume.

A propensity for aggregation was also observed for monodisperse native VLPs that had been correctly assembled in cells, and that had not been disassembled and reassembled in vitro. After storage for long enough at relatively high concentrations (e.g., 0.4 mg/mL) at either 25 °C or 4 °C in PBS pH = 7.6, intact, free VLPs formed aggregates similar to those observed after VLP reassembly in the same buffer. VLP aggregation was generally observed, irrespective of the buffer in which the particles were stored or reassembled. Those buffers included phosphate-based buffers from pH 6.0 to 8.0 containing from 0.15 to 2.0 M NaCl, and 75 mM Tris-HCl pH = 6.8, 250 mM NaCl. The stickiness of native MVM VLPs led also to the clustering of latex beads, due to adsorption of VLPs to the beads and to each other (Supplementary Figure S2). The above evidence indicated that correct VLPs are reassembled first, but that a fraction of them that increased with initial capsid protein concentration can later aggregate to form VLP clusters of different sizes.

### 3.3. Effect of Macromolecular Crowding on VLP Reassembly Efficiency

A test was then carried out to see whether the reassembly efficiency could be increased by using a macromolecular crowding agent to increase the capsid protein (VP2) chemical activity (“effective concentration”) without actually increasing the number of protein molecules in the sample volume. Ficoll-70 was chosen as the crowding agent because of its low viscosity, a size comparable to those of many proteins, and its low tendency to interact specifically with proteins and other solutes [22].

**Figure 5:**
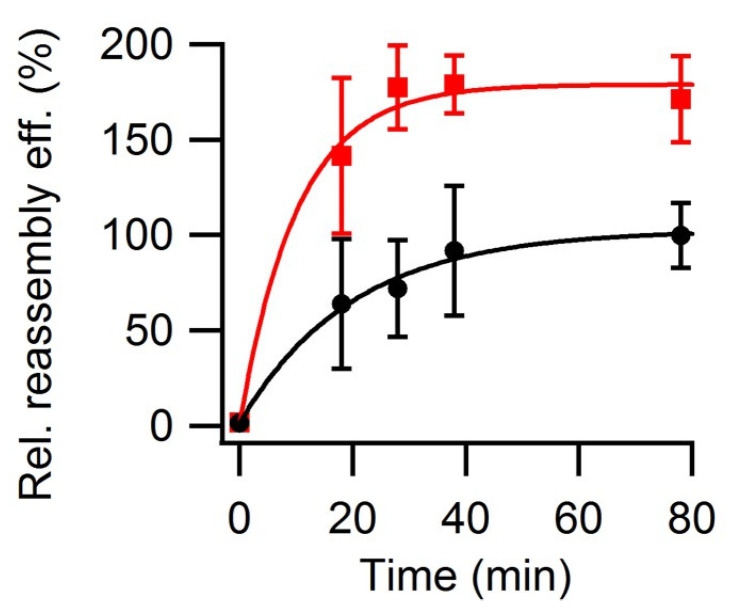
VLP reassembly efficiency as a function of time in the presence or absence of macromolecular crowding conditions. Reassembly of VLPs was performed at 25 °C in PBS pH = 7.6 using a protein concentration of 0.35 mg/mL, either in the absence (black circles) or presence (red squares) of 50 gr/L Ficoll-70. Relative reassembly efficiency values are given, using as a reference (100%) the efficiency obtained at the end of the reaction (*t* = 80 min) in the absence of Ficoll-70. Vertical bars represent standard deviations. The data were fitted to exponential curves (continuous lines).

The relative reassembly efficiency in the presence or absence of 50 g/L Ficoll-70 at 25 °C in PBS (pH = 7.6) was compared as a function of reaction time (Figure 5). The results showed that, under these conditions, macromolecular crowding increased both the reassembly rate and the reassembly efficiency (about 2-fold). The increase in reassembly efficiency was limited, however, because macromolecular crowding also increased the fraction of aggregated VLPs, as expected from the increase in the chemical activity of VP2 under such conditions.

### 3.4. Effect of Temperature on VLP Reassembly Efficiency

**Figure 6:**
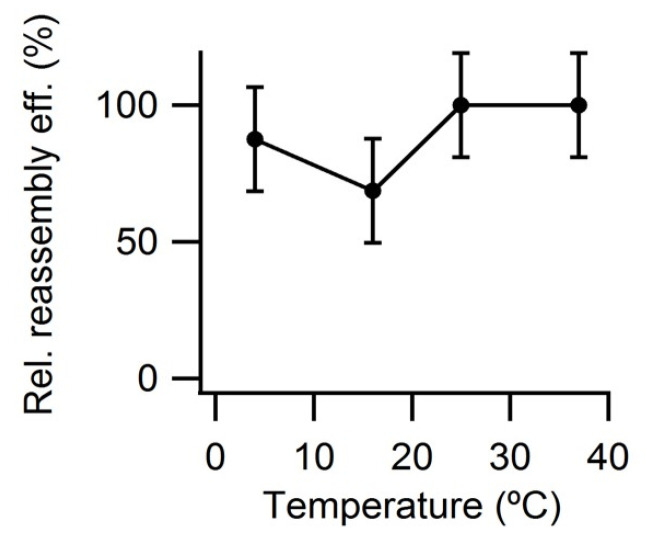
VLP reassembly efficiency as a function of temperature. Reassembly of VLPs was performed at 25 °C in PBS pH = 7.6, using a protein concentration of 0.6 mg/mL. Relative reassembly efficiency values after the reaction was complete (*t* = 2.5 h) are given, using as a reference (100%) the efficiency obtained at 25 °C. Vertical bars represent standard deviations.

Next, tests were carried out to see whether the reassembly efficiency could be increased by using temperatures lower or higher than 25 °C. After the reassembly reaction was essentially complete (the VLP yield reached a plateau) the reassembly efficiency was similar at 25 °C or 37 °C, and somewhat lower at 4 °C or 15 °C (Figure 6).

### 3.5. Effect of Ionic Strength on VLP Reassembly Efficiency

**Figure 7:**
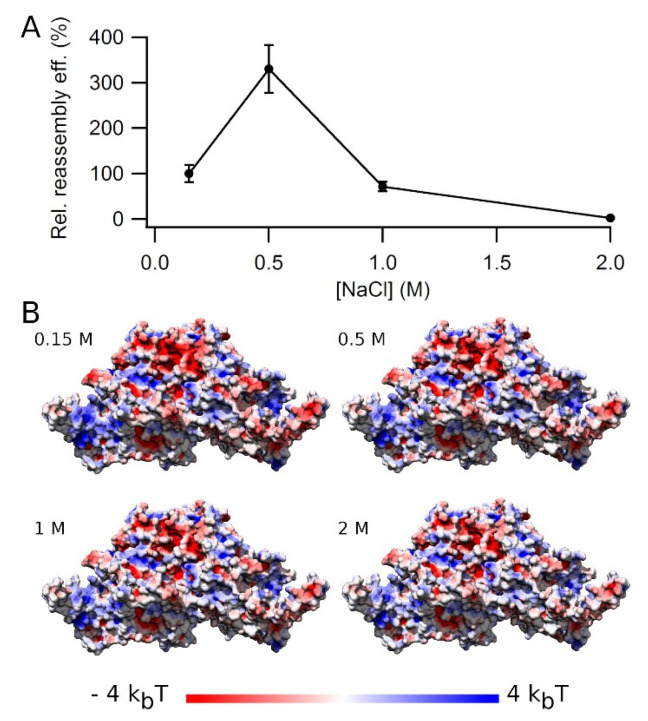
VLP reassembly efficiency as a function of ionic strength. (**A**) Reassembly of VLPs was performed at 25 °C in PBS pH = 7.6, using a protein concentration of 0.65 mg/mL and increasing amounts of NaCl, from 0.15 M to 2 M. Relative reassembly efficiency values after the reaction was complete (*t* = 80 min) are given, using as a reference (100%) the efficiency obtained at physiological ionic strength (0.15 M NaCl). Vertical bars represent standard deviations. (**B**) Calculated electrostatic potentials represented on the MVM capsid trimer surface as a function of ionic strength. The value of the electrostatic potential is expressed in *k_B_T* units (where *k_B_* is the Boltzmann constant and *T* the temperature) and color coded from red (negative) to blue (positive) in the trimer structure viewed from the side.

Electrostatic screening of protein–protein interactions by increasing the ionic strength may heavily influence interactions between capsid proteins and/or VLP aggregation. Thus, the effect of ionic strength on VLP reassembly efficiency was tested by increasing the NaCl concentration in the buffer from 0.15 M up to 2 M (Figure 7A). The reassembly efficiency was strongly dependent on the ionic strength, reaching a maximum around 0.5 M NaCl (3-fold the reassembly efficiency obtained at 0.15 M NaCl).

We then asked whether the higher reassembly efficiency found at 0.5 M NaCl correlated with some change in the values and distribution of the surface electrostatic potential on the VLP surface, including the intertrimer interfaces. Simple surface electrostatic calculations (Figure 7B and Video S1) revealed ionic strength-dependent changes at many locations of the trimer surface, including the intertrimer interfaces, especially at 1–2 M NaCl when compared to 0.15–0.5 M NaCl. However, no specific local change in electrostatic potential could be identified as responsible for the high increase in reassembly efficiency at 0.5 M NaCl, relative to lower or higher salt concentrations.

### 3.6. Effect of pH on VLP Reassembly Efficiency

**Figure 8:**
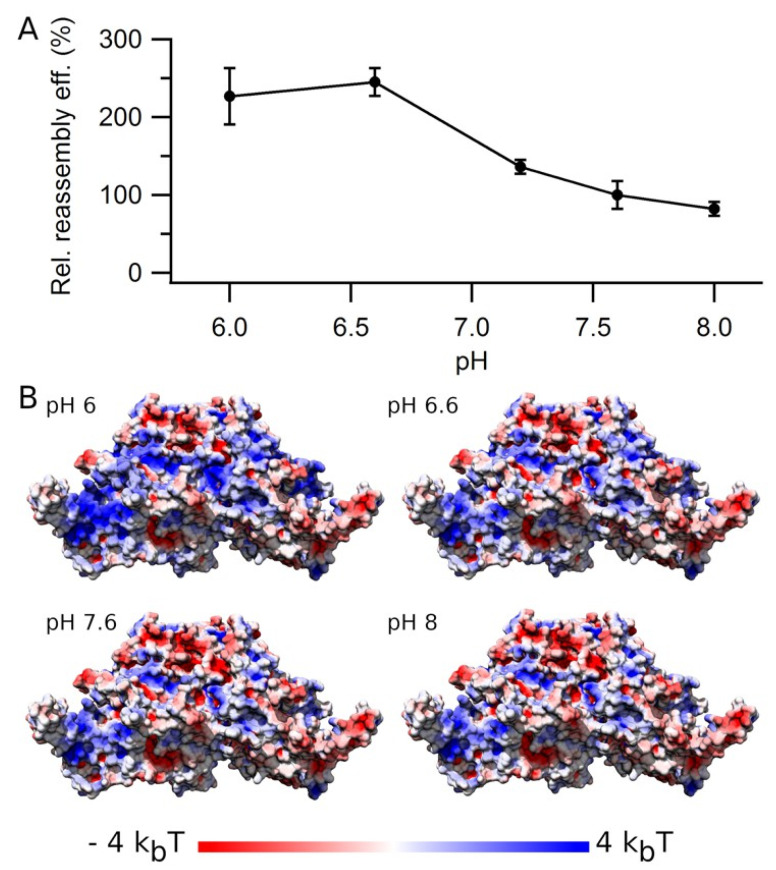
VLP reassembly efficiency as a function of pH. (**A**) Reassembly of VLPs was performed at 25 °C in PBS using a protein concentration of 0.6 mg/mL at different pH from 6.0 to 8.0). Relative reassembly efficiency values after the reaction was complete (*t* = 6 h) are given, using as a reference (100%) the efficiency obtained at pH = 7.6. Vertical bars represent standard deviations. (**B**) Calculated electrostatic potentials represented on the MVM capsid trimer surface as a function of pH. The value of the electrostatic potential is expressed in *k_B_T* units (where *k_B_* is the Boltzmann constant and *T* the temperature) and color coded from red (negative) to blue (positive) in the trimer structure viewed from the side.

Changes in the protonation state of acidic or basic side chains on the VLP surface or at the intertrimer interfaces could also influence VP2–VP2 interactions and/or VLP aggregation. Thus, we compared the reassembly efficiency within a pH interval from 6 to 8 (Figure 8A). The reassembly efficiency was clearly pH-dependent, being 2- to 3-fold higher at slightly acidic pH (6.0–6.6) than at neutral or slightly basic pH (7.2–8.0).

**Figure 9:**
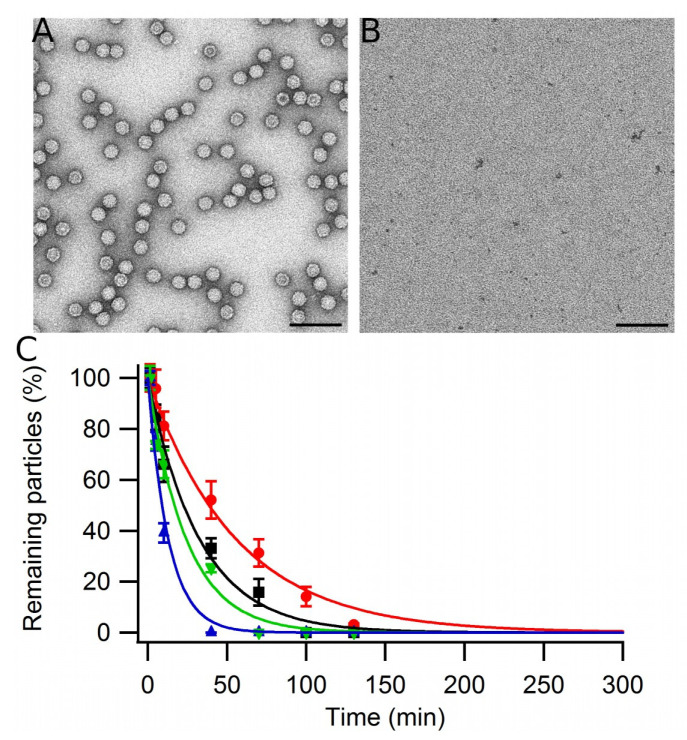
VLP stability as a function of pH. VLPs at a final concentration of 0.05 mg/mL were disassembled at 25 °C using GdmHCl at a final concentration of 3.75 M. (**A**,**B**) Electron micrographs of a VLPs before starting the experiment (**A**) or after complete dissociation using GdmHCl (**B**). Scale bars in A and B correspond to 100 nm. (**C**) Average percent VLPs remaining after incubation for different times in different buffers: (i) 150 mM Tris-HCl, 650 mM NaCl at pH = 7.6 (blue triangles) or pH = 6.8 (green inverted triangles); (ii) PBS at pH = 6.2 (black squares) or pH = 5.5 (red circles). Vertical bars represent standard deviations. The values were fitted to simple exponential decays (continuous lines) that correspond to the dissociation of the VLPs into their constituent subunits.

The stability of many viral capsids is pH-dependent. Thus, a higher capsid stability at slightly acidic pHs could contribute to the higher reassembly efficiency determined for MVM VLP at acidic pH, compared to neutral or slightly basic pH. To test the pH dependency of VLP stability, a dissociating agent was added (3.75 M GdmHCl), and the disassembly rate of purified VLPs as a function of pH was compared in two different buffers with a different ionic strength (Figure 9). The VLPs dissociated more slowly as the pH was lowered from 7.6 to 6.8 in a high salt (0.65 M NaCl) Tris-HCl buffer, or from 6.2 to 5.5 at physiological ionic strength (0.15 M NaCl) in PBS buffer. Thus, VLP stabilization could contribute to explaining the higher assembly efficiencies determined at slightly acidic pHs.

As for ionic strength, we asked whether the higher reassembly efficiency and particle stability at slightly acidic pH correlated with some change in the values and distribution of the surface electrostatic potential on the VLP surface, including the intertrimer interfaces. Surface electrostatic calculations (Figure 8B and Video S2) revealed pH-dependent changes on many parts of the trimer surface, including the intertrimer interfaces at pH = 6.0–6.6 when compared to pH = 7.2–8.0, which could be related to the higher reassembly efficiency. Which one(s) of those local variations in electrostatic potential is (are) responsible for the observed changes in reassembly efficiency as a function of pH or ionic strength remains to be investigated in more detail.

### 3.7. Combined Effects of Ionic Strength and pH on VLP Reassembly Efficiency

**Figure 10:**
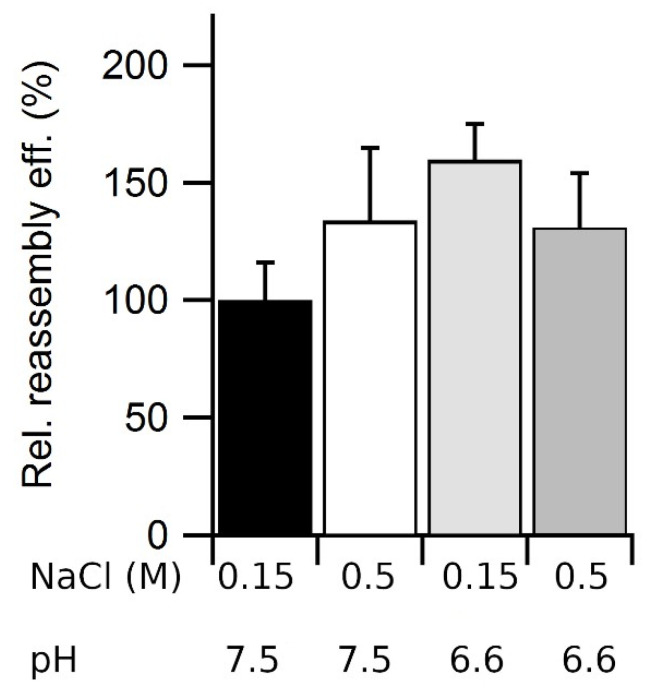
Combined effects of ionic strength and pH on VLP reassembly efficiency. Reassembly of VLPs was performed in PBS at 25 °C using a protein concentration of 0.05 mg/mL and the following pH and ionic strengths: pH = 7.6, 0.15 M NaCl (black bar); pH 7.6, 0.5 M NaCl (white bar); pH 6.6, 0.15 M NaCl (light grey bar); and pH 6.6, 0.5 M NaCl (dark grey bar). Relative reassembly efficiency values after the reaction was complete (*t* = 3 h) are given, using as a reference (100%) the efficiency obtained at pH = 7.6 and 0.15 M NaCl. Error bars represent propagated errors.

We then asked whether the increases in reassembly efficiency at relatively high ionic strength or acidic pH relative to physiologic ionic strength or neutral or basic pH were additive. The reassembly efficiency in PBS was compared at pH = 7.6, 0.15 M NaCl, pH 7.6, 0.5 M NaCl; pH 6.6, 0.15 M NaCl; and pH 6.6, 0.5 M NaCl (Figure 10). As expected from the experiments described above, both a higher ionic strength and a slightly acidic pH alone increased the reassembly efficiency, but their combined effect was not higher than that of either condition tested separately. This result could be expected, as any effect on assembly efficiency due to local changes in electrostatic charge due to a pH change would depend on the amount of electrostatic screening, which depends on the ionic strength.

### 3.8. Dissociation of VLP Aggregates

Variations in some conditions tested for MVM VLP assembly, including capsid protein concentration, the absence or presence of a macromolecular crowding agent, ionic strength, or pH, led to 2- to 3-fold increases in reassembly efficiency. However, the maximum absolute reassembly efficiencies thus achieved were never higher than 35–40%. From these observations alone it could be thought that, in vitro, the self-assembly reaction that builds the MVM capsid is only moderately efficient, and not fully reversible. In fact, incomplete or aberrant MVM VLPs [38] were only very rarely observed under any tested condition. Moreover, as already described above, the reassembly efficiency was limited not because the assembly process itself was inefficient, but because a fraction of the correctly reassembled VLPs became aggregated and were not counted; the same aggregation was observed during storage of native VLPs directly obtained from cells.

Different conditions were tested to attempt the disaggregation of the VLP clusters. Sonication did not achieve any substantial effect. Addition of a chaotropic agent (GdmHCl) at moderate concentrations (1.75 M) led to some disaggregation of large clusters into smaller clusters and individual free VLPs visualized by TEM, which again confirmed that the aggregates are made of fully reassembled VLPs. However, the addition of GdmHCl not only led to the release of some VLPs from the aggregates, but also to the dissociation of some of the free VLPs in the sample. Thus, the yields of free VLPs were not significantly increased (results not shown). Monodisperse suspensions of reassembled VLPs of MVM could be achieved only by elimination of the remaining aggregates through centrifugation.

### 3.9. Effect of Conditions on Assembly Fidelity

Extensive inspection using EM of the products of MVM VLP reassembly in vitro during this study did not reveal significant amounts of incorrectly assembled, aberrant viral particles under any tested condition of protein concentration, macromolecular crowding, temperature, pH, or ionic strength. These results provide evidence for a remarkable fidelity of MVM assembly that is virtually unaffected by changing conditions.

## 4. Discussion

The assembly of MVM and other parvoviruses inside infected mammalian cells is a complex process [25,50]. Stable trimers of MVM VPs are formed in the cytoplasm and translocated into the nucleus in response to specific nuclear localization signals [49,51,52]. In the nucleus, the VP trimers are made competent for assembly into capsids by undergoing a conformational change [49,53]. Efficient capsid assembly may be triggered by VP phosphorylation [54], and also requires the viral NS2 polypeptide [55]. Likewise, an assembly-activating protein was essential for nuclear assembly of the capsid of at least another parvovirus, the adeno-associated virus (AAV) [56]. Once the MVM capsid is assembled, the viral ssDNA genome is packaged through one of the capsid pores [57].

Recombinant VP2-only capsids (VLPs) of MVM and other parvoviruses (canine parvovirus (CPV) and porcine parvovirus) have been assembled in insect cells [27,37,58,59]. The atomic structure of the MVM VLP was indistinguishable from that of the authentic capsid [44]. However, parvovirus capsid assembly in insect cells occurred not in the nucleus, but in the cytoplasm, and was an inefficient process in most cases, perhaps because of the lack of adequate VP phosphorylation [37,53,58] and/or the absence of specific auxiliary proteins.

Recombinant VLPs of MVM, AAV2 or human parvovirus B19 have been assembled in vitro starting from VP subunits [38,60,61]. During the in vitro assembly of AAV2 VLPs, the yield of structurally correct capsids was low, and capsid fragments or aberrant forms were abundant, even in the presence of assembly-promoting factors [60]. In vitro assembly of B19 VLPs was efficient in PBS at neutral pH, but was highly sensitive to changes in pH, ionic strength or temperature. Those changes led to low numbers of free, correctly assembled VLPs, mixed with abundant capsid fragments and or heterogeneous aggregates, and many free VP molecules remained unassembled [61].

In vitro assembly of the MVM capsid along a defined pathway [38] did not require any auxiliary viral or cellular protein, which indicates that the information needed for this morphogenetic process is entirely contained in the VP2 structure. This conclusion is in line with the general physical principles invoked for protein folding and for the self-assembly of viral capsids [62,63] and other protein complexes. However, the efficiency and fidelity of MVM capsid self-assembly in vitro and the conditions that could affect this process had not previously been evaluated. The results obtained in the present study revealed that: (i) under very different conditions, the in vitro assembly of MVM VLPs is a remarkably faithful process in which no, or very few, aberrant particles are formed; (ii) MVM VLPs can be disassembled and efficiently reassembled in vitro; (iii) the natural tendency of MVM VLPs to aggregate at high-enough protein concentration reduced the yield of free VLPs; however, under specific conditions of protein concentration, macromolecular crowding, ionic strength and pH, yields of free VLPs (disregarding aggregates) reached up to ~40% of the possible maximum.

The above observations reinforce the notion that the higher complexity of parvovirus capsid assembly in mammalian cells arose because of the need to achieve during infection a regulated, highly efficient virus morphogenetic process in a compartmentalized, intricate cellular environment. In a highly dilute solution in the test tube under adequate conditions, the isolated, stable VP trimers were able to spontaneously and efficiently recognize each other with the correct orientation. The result is the faithful and efficient self-assembly of the capsid through a defined pathway ([38], and this study). The process is comparatively robust, in that changes in VP concentration, macromolecular crowding, temperature, pH, or ionic strength did not significantly affect its fidelity. However, the VLPs themselves had a tendency to aggregate, and even moderate increases in VP concentration or macromolecular crowding resulted in increased VLP aggregation, reducing the yield of free VLPs. In the highly crowded environment of an infected cell where many VP copies are being produced, efficient MVM capsid assembly may require precise morphogenetic control, including auxiliary factors [55,56] acting as chaperones that disfavor non-productive assembly pathways.

To sum up, this study has defined conditions for the faithful and efficient in vitro self-assembly of MVM VLPs in the absence of additional factors that may be required for in vivo capsid assembly. These results encourage the use of MVM VLPs as nanocarriers for different compounds. It must be noted that the defined assembly conditions constitute a starting point that might have to be changed somewhat, depending on the influence that the desired cargo molecules could have on the intertrimer interactions. The high thermal, chemical and mechanical stability of these virus-based VLPs and their biological features, such as their nuclear tropism, make them suitable choices for targeted drug delivery or other biomedical or nanotechnological applications.

## Data Availability

Data are available from the corresponding authors upon request.

## Supplementary Materials

The following supporting information can be downloaded at https://www.mdpi.com/article/10.3390/v15051054/s1, Figure S1: VLP counts by TEM as a function of the dilution of a concentrated VLP suspension; Figure S2: clustering of latex beads through adsorption of MVM VLPs to the beads and to each other; Video S1: calculated electrostatic potentials represented on the MVM capsid trimer surface as a function of ionic strength; Video S2: calculated electrostatic potentials represented on the MVM capsid trimer surface as a function of pH.
