# Supplementary material for: Electrostatic Screening, Acidic pH and Macromolecular Crowding Increase the Self-Assembly Efficiency of the Minute Virus of Mice Capsid In Vitro
Source: Viruses. 2023 Apr 25;15(5):1054. doi: 10.3390/v15051054 (PMC10224108; doi:10.3390/v15051054)
Supplement: Supplementary file 1 [file viruses-15-01054-s001.zip › viruses-2326868-supplementary/Fuertes et al Sup Mat.pdf]

Supplementary Material

Electrostatic Screening, Acidic pH and Macromolecular Crowding Increase the Self-Assembly Efficiency of the Minute Virus of Mice Capsid *in Vitro*

Miguel Angel Fuertes, Diego López Mateos, Luis Valiente, Alicia Rodríguez Huete, Alejandro Valbuena* and Mauricio G. Mateu*

Centro de Biología Molecular "Severo Ochoa", Universidad Autónoma de Madrid, Cantoblanco, 28049 Madrid, Spain

* Authors for correspondence. E-mail: mgarcia@cbm.csic.es; avalbuena@cbm.csic.es

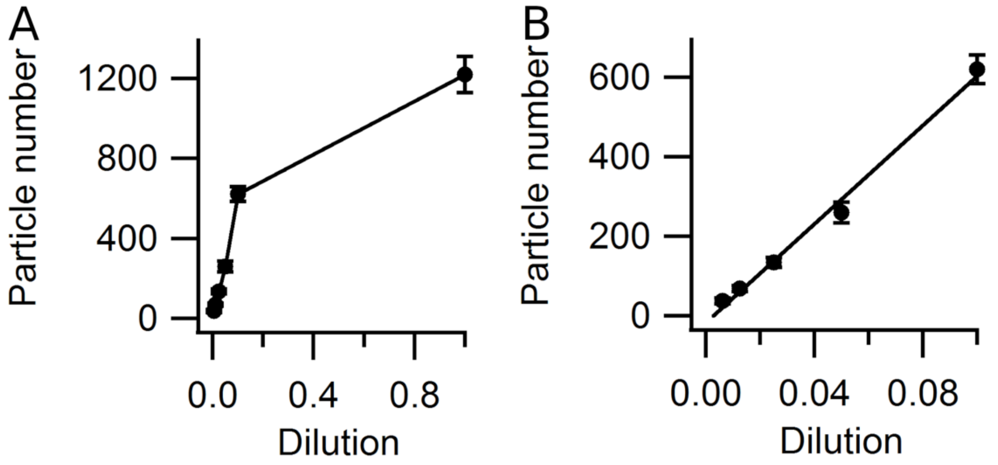

Figure S1. VLP counts by TEM as a function of the dilution of a concentrated VLP suspension. (A) full tested range, from 1/300 dilution to 1 (undiluted). (B) Linear part of the tested range, from 1/300 to 1/10 dilution (r^2=0.99).

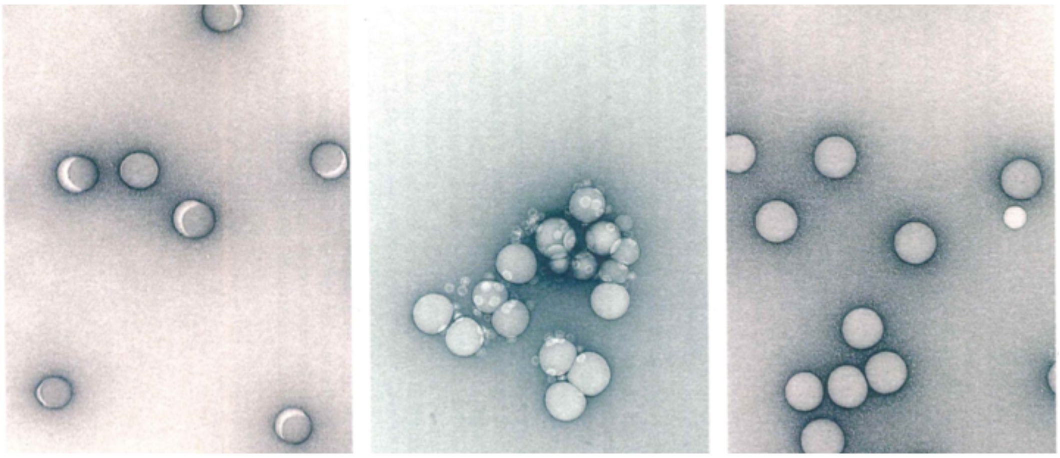

Figure S2. Clustering of latex beads through adsorption of MVM VLPs to the beads and to each other. (A) monodisperse latex beads. (B) clustered beads after addition of purified native VLPs; many VLPs can be seen adsorbed on the bead surface (C) monodisperse beads after dissociation of the VLPs by addition of 4M GdmHCl.

Video S1. Calculated electrostatic potentials represented on the MVM capsid trimer surface as a function of ionic strength.

Video S2. Calculated electrostatic potentials represented on the MVM capsid trimer surface as a function of pH.
